# OR11-002 - Mutations in MVK cause non-syndromic RP

**DOI:** 10.1186/1546-0096-11-S1-A191

**Published:** 2013-11-08

**Authors:** AM Siemiatkowska, M Stoffels, K Neveling, A Simon, PM van Hagen, AI den Hollander, FP Cremers, LI van den Born, RW Collin

**Affiliations:** 1 Department of Human Genetics, Radboud University Nijmegen Medical Centre, Nijmegen, Netherlands; 2 General Internal Medicine, Radboud University Nijmegen Medical Centre, Nijmegen, Netherlands; 3 Department of Immunology, Erasmus MC, University Medical Center Rotterdam, Rotterdam, Netherlands; 4 Department of Ophthalmology, Radboud University Nijmegen Medical Centre, Nijmegen, Netherlands; 5 The Rotterdam Eye Hospital, Rotterdam, Netherlands

## Introduction

Retinitis pigmentosa (RP) is a genetically heterogeneous retinal disease. Typically beginning with night blindness, RP is characterized by rod cell degeneration followed by cone cell death, which may ultimately lead to complete blindness. Despite extensive knowledge about genes involved in RP pathogenesis, in several cases the genetic cause remains elusive.

## Objectives

We aimed to identify novel genes that are involved in the etiology of RP.

## Methods

After detailed clinical characterization including funduscopy and optic coherence tomography, exome sequencing analysis was performed in a proband of Dutch origin with non-syndromic autosomal recessive RP. Identified mutations were tested for segregation within the family and in a large cohort of genetically unsolved RP patients. Upon identification of mutations in *MVK*, encoding mevalonate kinase (MK), patients with mutations in this gene underwent extensive clinical re-examination. MK enzyme activity was analyzed in cultured lymphoblastoid cells and mevalonic acid levels were measured in urine samples.

## Results

Exome variant filtering and prioritization led to the identification of compound heterozygous mutations in *MVK* (p.I268T and p.A334T) in the proband and her affected brother. Screening of 269 non-syndromic RP patients revealed an additional individual who was homozygous for the p.A334T alteration. Clinical re-evaluation of all three patients revealed a relatively classic form of RP with variable extra-ocular symptoms, such as history of recurrent childhood febrile crises in two, and mild ataxia in one patient. All three affected individuals showed a significantly decreased mevalonate kinase activity and strongly elevated levels of urinary mevalonic acid.

## Conclusion

Although the MK activity in cells and mevalonic acid concentrations in urine are strongly aberrant as in patients with systemic mevalonate kinase deficiencies (MKD), only mild clinical symptoms related to these phenotypes are observed in our patients, who were initially classified to have non-syndromic RP. Herewith, we add another phenotype to the spectrum of diverging disorders associated with mutations in *MVK*.

## Competing interests

None declared.

